# Fluorine-Containing Dibenzoanthracene and Benzoperylene-Type Polycyclic Aromatic Hydrocarbons: Synthesis, Structure, and Basic Chemical Properties

**DOI:** 10.3390/molecules23123337

**Published:** 2018-12-16

**Authors:** Otohiro Gotsu, Tomomi Shiota, Hiroki Fukumoto, Tomoko Kawasaki-Takasuka, Takashi Yamazaki, Tomoko Yajima, Tomohiro Agou, Toshio Kubota

**Affiliations:** 1 Department of Quantum Beam Science, Graduate School of Science and Engineering, Ibaraki University, 4-12-1 Nakanarusawa, Hitachi, Ibaraki 316-8511, Japan; 16nd105r@vc.ibaraki.ac.jp (O.G.); tomo.514a@gmail.com (T.S.); 2 Division of Applied Chemistry, Institute of Engineering, Tokyo University of Agriculture and Technology, 2-24-16 Nakamachi, Koganei, Tokyo 184-8588, Japan; takasuka@cc.tuat.ac.jp (T.K.-T.); tyamazak@cc.tuat.ac.jp (T.Y.); 3 Department of Chemistry, Faculty of Science, Ochanomizu University, Otsuka, Bunkyo-ku, Tokyo 112-8610, Japan; yajima.tomoko@ocha.ac.jp

**Keywords:** polycyclic aromatic hydrocarbons, dibenzoanthracene, benzoperylene, π-conjugated molecules, Mallory reaction, addition-defluorination, octafluorocyclopentene

## Abstract

Intramolecular photocyclization of stilbene derivatives (Mallory reaction) is one of the efficient methods for building polycyclic aromatic hydrocarbon (PAH) frameworks, and is also expected to be applicable to synthesis of fluorine-containing PAHs (F-PAHs). In this study, dibenzoanthracene-type (**4a**) and benzoperylene-type (**4b**) F-PAHs were synthesized using the Mallory reaction of the 1,4-distyrylbenzene-type π-conjugated molecule (**3a**), which was prepared by addition-defluorination of available octafluorocyclopentene (OFCP) and aryllithium in three steps. The structure of **4a** originating from π–π interaction was characterized by X-ray crystallographic analysis. The absorption maxima of UV-Vis spectra and emission maxima of photoluminescence spectra of the PAHs were positioned at a longer wavelength compared to those of the corresponding unsubstituted PAHs, presumably due to the electron-withdrawing nature of perfluorocyclopentene (PFCP) units. The effect of PFCP units in F-PAHs was also studied by time-dependent density functional theory (TD-DFT) calculation.

## 1. Introduction

Fluorine-containing polycyclic aromatic hydrocarbons (F-PAHs) [1] have attracted much attention in the field of material chemistry as n-type semiconductors for fabrication of electronic and optical devices [2]. In order to synthesize F-PAHs in short steps, it is inevitable to introduce fluorine or organofluorine groups into the PAH framework. Direct fluorination of PAH with a fluorinated reagent [3,4] is a typical and simple synthetic method, however it requires expensive XeF_2_ [3] or treatment of a fluorinated reagent (e.g., *N*-fluoro-2,4-dinitroimidazole) using F_2_ gas [4]. Furthermore, this method usually yields a mixture of regioisomers of F-PAHs, due to the difficulty of selective fluorination to the mother PAH skeleton, except for a few cases [5,6]. On the other hand, the ring-closing process of fluorine-containing aromatic compounds can basically achieve the desired regiospecific F-PAH. For example, Ichikawa and his coworkers reported metal-catalyzed Friedel–Crafts-type intramolecular cyclization of difluoroalkene and the nearest phenyl unit [7,8,9]. In this case, fluorine was pinpoint introduced to a phenacene skeleton. Kikuzawa and his groups developed a synthesis of hexafluoro-hexa-*peri*-hexabenzocoronene, which showed an n-type character in a fabricated FET (field effect transistor) device, using chemical oxidative intramolecular cyclization (Scholl reaction) of the corresponding hexaphenylbenzene [10].

The Mallory reaction is a useful intramolecular photocyclization of stilbene derivatives for synthesis of phenanthrenes via cis-trans isomerization [11,12,13], which is also applicable to the formation of F-PAHs. In recent years, regiospecific F-PAHs have been synthesized by photocyclization of 1,2-diarylfluoroalkenes via Julia−Kocienski olefination [14] or the Wittig reaction [15]. Our group also reported fluorine-containing phenanthrenes [16] using the Mallory reaction of the corresponding stilbene-type precursor, which were obtained by the reaction of available octafluorocyclopentene (OFCP) with two equivalents of aryllithium under milder conditions (addition-defluorination [17]). The merit of addition-defluorination toward fluorine-containing alkene is the stepwise introduction of two different aromatic rings to give an asymmetric diarylethene in only two steps. Therefore, a combination of the Mallory reaction and addition-defluorination has the ability to supply a wide variety of F-PAHs containing perfluorocyclopentene (PFCP) unit(s).

Herein, we report the synthesis of 1,4-distyrylbenzene-like π-conjugated molecule bearing PFCP units by reaction of available OFCP with aryllithium derivatives. We also describe fluorine-containing dibenzoanthracene and benzoperylene using the sequential Mallory reaction of the synthesized precursor.

## 2. Results

### 2.1. Synthesis of Fluorine-Containing Dibenzoanthracene *(**4a**)* and Benzoperylene *(**4b**)*

**Scheme 1:**
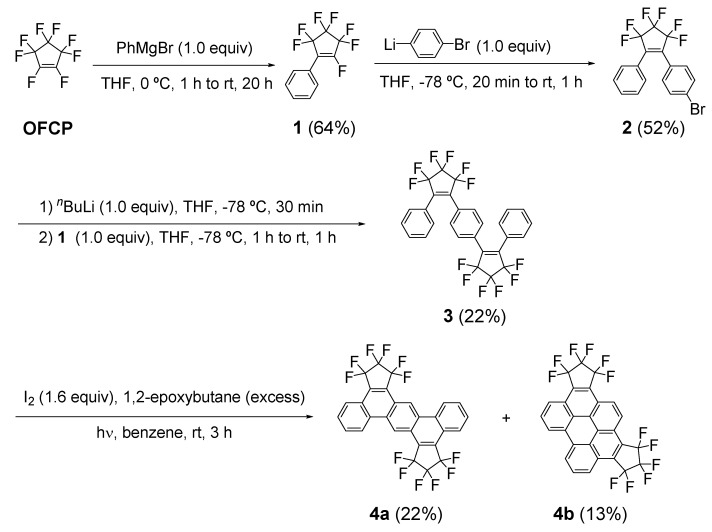
Synthetic route to fluorine-containing **4a** and **4b**.

The synthetic route to fluorine-containing dibenzoanthracene **4a** and benzoperylene **4b** is shown in Scheme 1. Preparation of 1-phenyl-heptafluorocyclopentene **1** (64%, literature 29% [17]) was carried out by reaction of OFCP and 1 equiv. of phenylmagnesium bromide, according to the literature [17]. Treatment of **1** with 4-bromophenyllithium in THF gave unsymmetric diarylethene **2** as a colorless oil in 52% yield. Lithiated **2**, generated from **2** and *^n^*BuLi, was reacted with **1** in THF to afford the precursor for the following Mallory reaction, giving **3** as white powder in 22% yield.

Mallory reaction of **3** in benzene was carried out under light irradiation (λ = 365 nm) at room temperature (rt) for 3 h in the presence of iodine (1.6 equiv.) as an oxidant and excess amounts of 1,2-epoxybutane as a scavenger of the formed hydrogen iodide to give the mixture of the ring-closed products **4a** and **4b**, confirmed by ^1^H-NMR spectroscopy. Benzoperylene-type **4b** in the reaction mixture was sublimed under reduced pressure (0.1 kPa) at 160 °C, however the sublimate still contained a small amount of dibenzoanthracene-type **4a**. The sublimate was purified by preparative layer chromatography (PLC) (silica) to afford pure **4a** and **4b** (13% yield). After removal of the first sublimate, the residue was heated up to 250 °C under 0.1 kPa to result in sublimation of **4a** (22% as total yield); the low yields were due to loss of the products in repeated purification (PLC and sublimation). Compared to the solubility of **3**, both **4a** and **4b** have quite low solubility in organic solvents, such as chloroform and dichloromethane. Formation of [5]helicene-type compound was not detected after the Mallory reaction. It is known that photocyclization of 1,4-distyrylbenzene derivatives preferably gives benzoperylene, [5]helicene, or both, whereas photocyclization affords dibenzoanthracene in low yields [13,18,19]. The above Mallory reaction of **3** indicates that formation of dibenzoanthracene-like structure, and rotation of one of the PFCP units to form benzoperylene-like structure, are competitive as the second photocyclization. It is also supposed that the third photocyclization spontaneously occurred to give benzoperylene-like **4b** once the second photocyclization proceeded in [5]helicene-type form.

^1^H-NMR spectra of **3**, **4a**, and **4b** are depicted in the Supporting Information. In the spectrum of **3** (Figure S4), signals of two terminal phenyl and central phenylene protons are observed in the typical aromatic region. For spectra of **4a** and **4b** (Figures S7 and S9), all the peaks clearly appear at a lower magnetic field than those observed in that of **3**, owing to the magnetic deshielding effect of the extended π-conjugated PAH framework. In particular, the singlet observed at 9.77 ppm in the spectrum of **4a** is assignable to the protons bound to the carbons of the central ring in the dibenzoanthracene framework, due to the induced effect originating from the neighborhood fluorines in the PFCP units.

### 2.2. Molecular Structures of ***3***, ***4a***, and ***4b***

**Figure 1:**
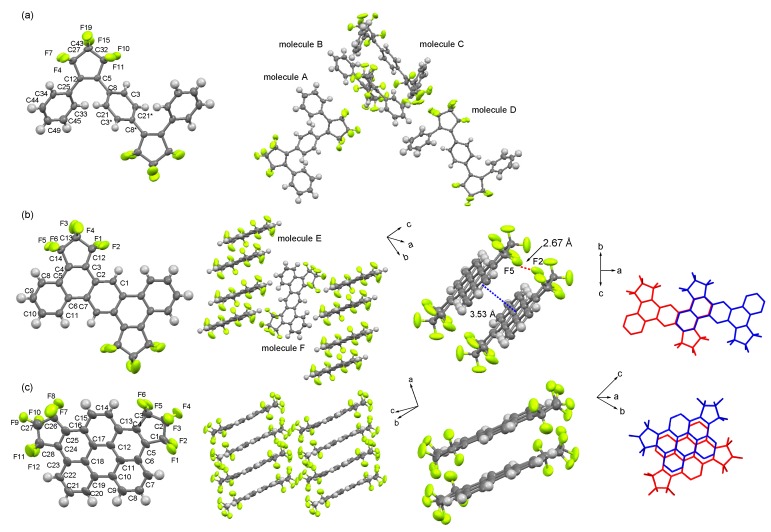
Molecular structures and packing modes of (**a**) **3**, (**b**) **4a**, and (**c**) **4b**. One of the independent molecules (**3**: molecule A, **4a**: molecule E) is shown for clarity. Two fluorine atoms (F9 and F10) bound to CF_2_ carbon (C27) in **4b** and some fluorine atoms in two independent molecules (molecules C and D) of **3** are disordered (see Figures S13 and S15). For **4a**, the face-to-face distance is calculated between the polyaromatic hydrocarbon (PAH) cores, which are optimized by the least square method. For **4b**, its crystallographic data are not satisfied to discuss the bond lengths and short-contacts distances (see Section 3.5).

The molecular structures of **3**, **4a**, and **4b** revealed by X-ray crystallographic study are shown in Figure 1. The structures of precursor **3** and dibenzoanthracene **4a** have four and two independent molecules in the unit cell, respectively. One of the independent molecules (molecule A for **3** in Figure 1a and molecule E for **4a** in Figure 1b) is depicted in Figure 1 for clarity. Benzoperylene **4b** (Figure 1c) and **3** (molecules C and D in Figure 1a) show disorder in fluorine atoms. Precursor **3** takes a flexible backbone with dihedral angle between the central phenylene plane (C3–C8–C21–C3*–C8*–C21*) and the terminal phenyl plane (C25–C34–C44–C49–C45–C33) of 60.2°, to result in no face-to-face π–π interactions between neighborhood molecules (Figure 1a). The C=C bond distance between C5 and C12 in the five-membered unit is 1.353(5) Å (molecule A), which is comparable to the reported similar diarylethene derivatives [20]. For **4a**, two independent molecules (molecules E and F) are herringbone-like packed in the unit cell, and one of two independent molecules (molecule E) partly overlaps to make a pair with terminal naphthalene units (C2-C3-C4-C5-C8–C9–C10–C11–C6–C7) in the neighboring molecule; the distance between the centroids in the dibenzoanthracene skeleton is 7.15 Å. The distance between dibenzoanthracene planes is 3.55 Å, due to the π–π interaction (Figure 1b). The shortest intermolecular fluorine–fluorine (F2-F5) distance between the pairing molecules is ca. 2.67 Å, which is shorter than the sum of the van der Waals radius (2.94 Å), indicating that not only the π–π interaction, but also the F–F interaction partly contribute to the packing of the molecules of **4a** [21]. The length of the C=C bond (C(3)–C(4)) fused with the perfluorocycloalkane unit is 1.354(5) Å. In the case of **4b**, the molecules construct a columnar structure extended to a axis in the crystal (Figure 1c). In sharp contrast to the case of **4a**, the nearest two molecules of **4b** fully overlap where possible, to avoid steric repulsion between the bulky PFCP units, Such overlap of benzoperilene skeletons is observed in unsubstituted benzoperylene [22] and 1,2-disubstituted benzoperylene [23].

### 2.3. UV-Vis and Photoluminescence Spectra of ***3***, ***4a***, and ***4b***

**Figure 2:**
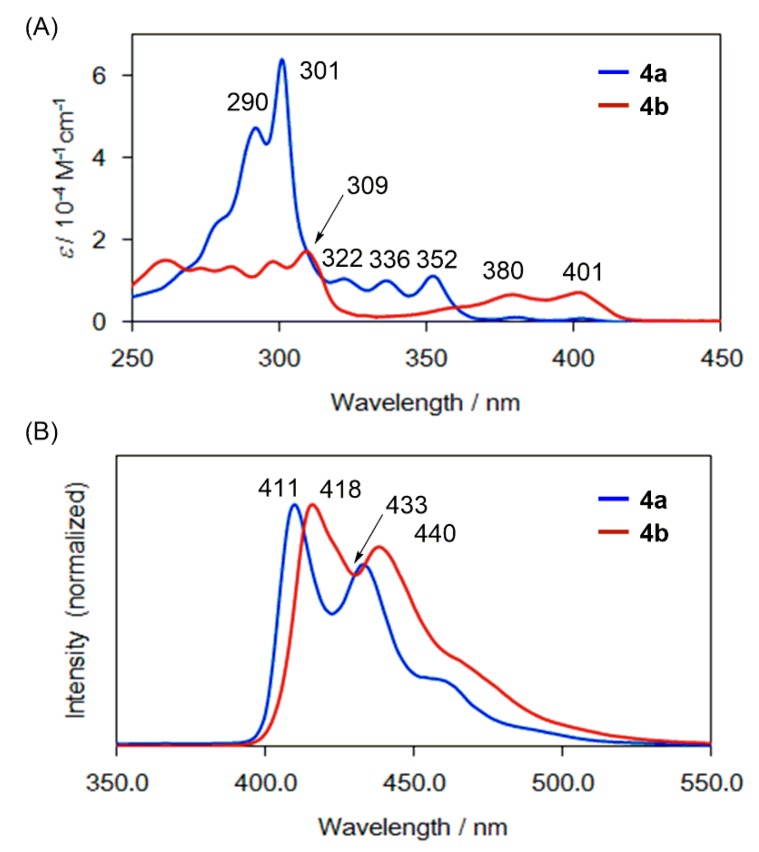
UV-Vis (**A**) and photoluminescence (**B**) spectra of **4a** (blue line) and **4b** (red line) in CH_2_Cl_2_.

UV-Vis and photoluminescence (PL) spectra of **4a** and **4b** are shown in Figure 2a,b, respectively, and the optical data are summarized in Table 1. For comparison, UV-Vis and PL spectra of the corresponding precursor **3** were also measured, as depicted in Figure S12. The absorption maximum (λ_max_) in the spectrum of **3** appears at 262 nm, due to localization of π-electrons along the flexible backbone of **3**. In contrast, the spectra of **4a** and **4b** in CH_2_Cl_2_ exhibit λ_max_ at 301 nm and 309 nm, respectively. This longer wavelength shift presumably originates from extension of the π-conjugated system on the molecules of **4a** and **4b**. The spectrum of dibenzoanthracene **4a** exhibits three medium peaks (ε = ca. 10,000 M^−1^∙cm^−1^) at 322, 336, and 352 nm, and two small peaks (ε = ca. 1000 M^−1^∙cm^−1^) around 390 nm, as comparable to those observed in unsubstituted dibenzoanthracene [24]. On the other hand, the spectrum of benzoperylene **4b** gives two clear peaks (ε = ca. 7000 M^−1^∙cm^−1^) around 380 and 400 nm, which is similar to those of the reported analogues [23]. Time-dependent density functional theory (TD-DFT) calculation supports assignments of absorption peaks in the UV-Vis spectrum of organic molecules. The TD-DFT calculation was carried out at the B3LYP/6-311G(d) level, and the initial structures of **4a** and **4b** were obtained using their X-ray crystallographic data. The results indicate that the bands that appeared at 401 and 309 nm in the spectrum of **4b** correspond to the transition from HOMO (the highest occupied molecular orbital) to LUMO (the lowest unoccupied molecular orbital) with oscillator strength (f) of 0.2041 (λ_calc_ = 399 nm), and the HOMO→LUMO + 1 (f = 0.3049, λ_calc_ = 307 nm) transition, respectively (Table S1). The DFT calculation also suggests that the HOMO→LUMO + 1 (f = 0.5972, λ_calc_ = 305 nm) transition corresponds to the large peak (ε = 64,000 M^−1^∙cm^−1^) at 301 nm in the spectrum of **4a**, and the weak band (ε = 1120 M^−1^ cm^−1^) around 380 nm is thought to be the HOMO→LUMO transition (f = 0.0146, λ_calc_ = 381 nm).

The Mallory reaction products are photoluminescent in CH_2_Cl_2_ (Figure 2b). The main PL peaks (λ_em_) of **4a** and **4b** appear at 411 and 418 nm, respectively. The λ_em_ peak positions of both **4a** and **4b** shift to longer wavelengths than those of the corresponding unsubstituted dibenzoanthracene (λ_em_ = 398 nm) [24] and benzoperylene (λ_em_ = 408 nm) [23], respectively. In recent years, it has been reported that introduction of electron-withdrawing methoxycarbonyl group(s) into the C1 and C2 carbons of benzoperylene causes a longer wavelength shift of their PL spectra [23]. In the case of **4b**, PFCP units may contribute to the red-shift of the λ_em_ peak, although the PFCP units are fused on the C3, C4, C11, and C12 carbons of **4b**. In contrast, the PL spectrum of precursor **3** exhibited a very weak band at approximately 430 nm in CHCl_3_ (Figure S12), due to the small effective π-conjugation length along the distorted molecule of **3**.

### 2.4. Computational Study

**Figure 3:**
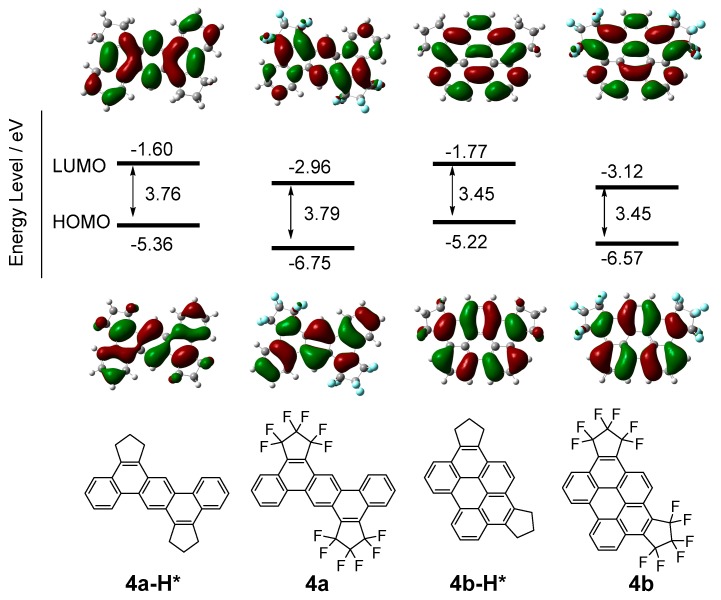
Molecular orbital diagram for fluorine-containing PAHs (F-PAHs) (**4a** and **4b**) and their corresponding PAH (4a-H* and 4b-H*), in which all fluorine atoms in **4a** and **4b** are replaced with hydrogen atoms.

To evaluate effect of the introduction of PFCP units to the PAH backbone, a computational study was carried out by DFT at the B3LYP/6-311G(d) level, together with 4a-H* and 4b-H*, in which all the fluorine atoms of **4a** and **4b** are replaced with hydrogen atoms for comparison (Figure 3). As shown in this diagram, the LUMOs in **4a** and **4b** are extended to the PFCP units, and their LUMO levels (−2.96 eV for **4a** and −3.12 eV for **4b**) are remarkably lower than those of 4a-H* (−1.60 eV) and 4b-H* (−1.77 eV). The HOMO levels of **4a** (−6.75 eV) and **4b** (−6.57 eV) are also lower than those of 4a-H* (−5.36 eV) and 4b-H* (−5.22 eV), due to electro-negative fluorine groups in PFCP units. The HOMO–LUMO gap of benzoperylene-type **4b** (3.45 eV) is smaller than that of dibenzoanthracene-type **4a** (3.79 eV). This trend is consistent with the difference in the UV-Vis absorption peaks between **4a** and **4b**, as described above.

## 3. Materials and Methods 

### 3.1. General

All manipulations were conducted by standard Schlenk techniques in argon atmosphere. ^1^H-, ^13^C-, and ^19^F-NMR spectra were measured with a Bruker AVANCE III 400 spectrometer (^1^H-NMR: 400 MHz, ^19^F-NMR: 376 MHz, ^13^C-NMR: 100 MHz; Bruker Corporation, Billerica, MA, USA). SiMe_4_ was used as an internal standard for ^1^H- and ^13^C-NMR spectra, and CFCl_3_ was adopted as an external standard for ^19^F-NMR spectra. UV-Vis and photoluminescence spectra were recorded on a Shimadzu UV-3100PC spectrometer (Shimadzu Corporation, Kyoto, Japan) and a Hitachi F-4500 spectrometer (Hitachi High-Technologies Corporation, Tokyo, Japan), respectively. Elemental analysis was carried out using a J-Science Lab JM10 microanalyzer (J-Science Lab Co., Ltd., Kyoto, Japan). High resolution mass spectra (HRMS) were taken using a JEOL JMS-700 analyzer (JEOL Ltd., Akishima, Japan). Mallory reaction of **3** was conducted using a USHIO Optical Modulex Multi-purpose lighting unit (irradiation wavelength = 365 nm, light power = 20 mW/cm^2^; USHIO INC., Tokyo, Japan). Octafluorocyclopentene (OFCP) was purchased and used without further purification. 1-Phenyl-heptafluorocyclopentene (**1**) was prepared according to the literature [17].

### 3.2. Synthesis of 1-(4-bromophenyl)-2-phenyl-3,3,4,4,5,5-hexafluorocyclopentene *(**2**)*

To a THF (10 mL) solution of 1,4-dibromobenzene (0.71 g, 3.0 mmol), ^n^BuLi (1.6 M hexane solution, 1.9 mL, 3.0 mmol) at −78 °C was added, and the reaction temperature was kept at −78 °C for 20 min. **1** (0.81 g, 3 mmol) was added to the reaction mixture at −78 °C, then the reaction temperature was raised up to rt for 1 h. The reaction mixture was poured into saturated NH_4_Cl aqueous solution (ca. 10 mL) and extracted with ethyl acetate (ca. 60 mL). The organic layer was washed with distilled water and dried over MgSO_4_. The solvent was removed at reduced pressure, and the resulting residue was purified by column chromatography on silica (eluent = hexane) to afford **2** as a colorless oil (0.63 g, 52%). ^1^H-NMR (400 MHz, CDCl_3_): δ 7.50–7.55 (m, 2H), 7.44–7.30 (m, 5H), 7.20 (d, *J* = 8.8 Hz, 2H). ^13^C{^1^H}-NMR (100 MHz, CDCl_3_): δ 140.26 (m), 138.50 (m), 132.26, 130.84, 130.54, 129.21, 129.06, 127.46, 126.66, 125.04, 116.33 (triplet of triplets (tt), *J* = 255.7 and 23.9 Hz), 111.07 (tt, *J* = 269.7 and 25.0 Hz). The central CF_2_ carbon in the five-membered ring was not clearly observed, presumably because the intensity of the peak with multicity became very weak. ^19^F-NMR (376 MHz, CDCl_3_): δ −110.38 (m, 2F), −110.60 (m, 2F), −131.66 (m, 2F). HRMS (FAB positive, *m*/*z*): Found, 406.9868; calcd. for C_17_H_10_BrF_6_ ([M + H]^+^), 406.9825.

### 3.3. Synthesis of 1,1′-[1,4-phenylenebis(3,3,4,4,5,5-hexafluorocyclopent-1-ene-2,1-diyl)]dibenzene *(**3**)*

To a THF (5 mL) solution of **2** (0.63 g, 1.5 mmol), ^n^BuLi (1.6 M hexane solution, 0.9 mL, 1.5 mmol) at −78 °C was added, and the reaction temperature was kept at −78 °C for 30 min. After consumption of **2**, **1** (0.41 g, 1.5 mmol) was added to the reaction mixture at −78 °C. The reaction temperature was kept at −78 °C for 1 h, and then raised up to rt for 1 h. The reaction mixture was poured into saturated NH_4_Cl aqueous solution (ca. 5 mL) and extracted with ethyl acetate (ca. 60 mL). The organic layer was washed with distilled water and dried over MgSO_4_. The solvent was removed at reduced pressure, and the resulting residue was purified by column chromatography on silica (eluent = hexane) to give **3** as white powder (0.19 g, 22%). ^1^H-NMR (400 MHz, (CD_3_)_2_CO): δ 7.58–7.45 (m, 10H), 7.43 (d, *J* = 7.6 Hz, 4H). ^13^C{^1^H}-NMR (100 MHz, (CD_3_)_2_CO): δ 141.33 (m), 138.87 (m), 130.83, 130.00, 129.63, 129.24, 129.08, 127.04, 116.42 (tt, *J* = 256.4 and 21.8 Hz), 111.29 (tt, *J* = 269.6 and 24.9 Hz). The central CF_2_ carbon in the five-membered ring was not clearly observed, presumably because the intensity of the peak with multicity became very weak. ^19^F-NMR (376 MHz, (CD_3_)_2_CO): δ −110.04 to −110.07 (m, 4F), −110.37 to −111.41 (m, 4F), −132.36 (quint, *J* = 4.5 Hz, 4F). Found: C, 58.17; H, 2.68. Calcd. for C_28_H_14_F_12_: C, 58.14; H, 2.44.

### 3.4. Synthesis of 1,1,2,2,3,3,9,9,10,10,11,11-dodecafluoro-1,2,3,9,10,11-hexahydrobenzo[k]dicyclopenta[f,m]tetraphene *(**4a**)* and 1,1,2,2,3,3,10,10,11,11,12,12-dodecafluoro-1,2,3,10,11,12-hexahydrobenzo[pqr]dicyclopenta[b,n]perylene *(**4b**)*

To a mixture of **3** (0.042 g, 0.073 mmol) and benzene (30 mL), iodine (0.029 g, 0.12 mmol) at rt was added in Ar atmosphere. After stirring for 30 min, 1,2-epoxybutane (0.3 mL, 3.5 mmol) was added to the mixture, which was stirred under light irradiation at rt for 3 h. The reaction mixture was poured into saturated sodium thiosulfate aqueous solution (10 mL), and the resulting organic layer was washed with distilled water (10 mL) and brine (10 mL). After separation and drying of the layer, the solvent was evaporated at reduced pressure. The resulting residue was heated under 0.1 kPa at 160 °C, and the sublimate was separated by column chromatography on silica (eluent: ethyl acetate/hexane = 1/4) to afford **4a** and **4b**. The residue after the first sublimation was heated again under 0.1 kPa at 250 °C to sublime **4a**. **4a**—colorless powder (10 mg, 22%). ^1^H-NMR (400 MHz, CDCl_3_): δ 9.77 (s, 2H), 9.01 (d, *J* = 8.0 Hz, 2H), 8.49 (d, *J* = 7.2 Hz, 2H), 8.07 (t, *J* = 7.2 Hz, 2H), 7.93 (t, *J* = 8.0 Hz, 2H). ^19^F-NMR (376 MHz, CDCl_3_): δ −105.45 (m, 4F), −106.06 (m, 4F), −128.75 (quint, *J* = 3.8 Hz, 4F). HRMS (FAB positive, *m*/*z*): Found, 574.0552; Calcd. for C_28_H_10_F_12_ ([M]^+^), 574.0591. **4b**—colorless powder (6 mg, 13%). ^1^H-NMR (400 MHz, CDCl_3_): δ 9.34 (d, *J* = 8.0 Hz, 2H), 9.05 (s, 2H), 8.83 (d, *J* = 8.0 Hz, 2H), 8.35 (t, *J* = 8.0 Hz, 2H). ^19^F-NMR (376 MHz, CDCl_3_): δ −105.61 (m, 4F), −106.17 (m, 4F), −128.7 (t, *J* = 3.8 Hz, 4F). HRMS (FAB positive, *m*/*z*): Found, 572.0458; Calcd. for C_28_H_8_F_12_ ([M]^+^), 572.0434.

### 3.5. X-Ray Crystallographic Analysis of ***3***, ***4a***, and ***4b***

Single crystals of **3**, **4a**, and **4b** suitable for X-ray crystallographic analysis were obtained by slow evaporation of the saturated CH_2_Cl_2_ (for **4a** and **4b**) or hexane (for **3**) solution at rt. The crystals were mounted on a Rigaku XtaLabMini diffractometer (Rigaku Corporation, Akishima, Japan) using Mo Kα radiation (λ = 0.71073 Å). Processing of the collected reflection data was carried out using the CrysAlisPro program (version 1.171.38.46, Rigaku Corporation, Akishima, Japan) [25]. The structure was solved by a direct method (SHELXT-2014/5) and refined by the full-matrix least square method on F^2^ for all reflections (SHELXTL-2014/7) [26]. All hydrogen atoms were placed using AFIX instructions, and all the other atoms were refined anisotropically. Precursor **3** and benzoperylene **4b** showed disorder in fluorine atoms (F25, F26, F27, F28, in **3**; F9 and F10 in **4b**). Dibenzoanthracene **4a** and **3** have two and four independent molecules in the unit cell, respectively. Crystallographic data of **3**, **4a**, and **4b** are deposited on the system of The Cambridge Crystallographic Data Centre: CCDC-1822475 (for **3**), CCDC-1822473 (for **4a**), and CCDC-1822474 (for **4b**). These crystal data can be obtained free of charge from the center via https://www.ccdc.cam.ac.uk/structures/.

Crystal data for **3**: C_28_H_14_F_12_, FW 578.39, –100 °C, 0.130 × 0.120 × 0.120 mm^3^, Triclinic, P-1, a = 10.1230(5) Å, b = 13.9273(9) Å, c = 18.2364(11) Å, V = 2414.6(3) Å^3^, α = 70.087(6)°, β = 88.323(5)°, γ = 87.402(5)°, Z = 4, D_calcd_ = 1.591 g cm^−3^, μ = 0.157 mm^−1^, F(000) = 1160, 2.014° ≤ θ ≤ 25.500°, reflection collected 21,605, independent reflections 8993 (R_int_ = 0.0373), completeness to θ_max_ 100.0%, data/restraints/parameters 8993/48/793, GOF on F^2^ 1.027, R_1_ [I > 2σ(I)] 0.0710, wR^2^ (all data) 0.2046, largest diffraction peak and hole 0.871 and −0.464 e Å^−3^.

Crystal data for **4a**: C_28_H_10_F_12_, FW 574.36, −100 °C, 0.230 × 0.180 × 0.150 mm^3^, Triclinic, P-1, a = 7.1452(7) Å, b = 9.4617(7) Å, c = 17.8343(15) Å, V = 1088.81(18) Å^3^, α = 79.109(7)°, β = 79.718(8)°, γ = 67.831(8)°, Z = 2, D_calcd_ = 1.752 g cm^−3^, μ = 0.174 mm^−1^, F(000) = 572, 2.342° ≤ θ ≤ 25.492°, reflection collected 9765, independent reflections 4065 (R_int_ = 0.0508), completeness to θ_max_ 99.9%, data/restraints/parameters 4065/0/361, GOF on F^2^ 1.031, R_1_ [I > 2σ(I)] 0.0634, wR^2^ (all data) 0.1985, largest diffraction peak and hole 0.365 and −0.357 e Å^−3^.

Crystal data for **4b**: C_28_H_8_F_12_, FW 572.34, −100 °C, 0.210 × 0.080 × 0.050 mm^3^, Triclinic, P-1, a = 7.438(3) Å, b = 11.078(3) Å, c = 13.181(3) Å, V = 1024.6(6) Å^3^, α = 72.22(3)°, β = 82.50(3)°, γ = 89.76(3)°, Z = 2, D_calcd_ = 1.855 g cm^−3^, μ = 0.184 mm^−1^, F(000) = 568, 1.638° ≤ θ ≤ 25.495°, reflection collected 9075, independent reflections 3811 (R_int_ = 0.1310), completeness to θ_max_ 100.0%, data/restraints/parameters 3811/54/379, GOF on F^2^ 1.058, R_1_ [I > 2σ(I)] 0.1448, wR^2^ (all data) 0.4404, largest diffraction peak and hole 0.963 and −0.676 e Å^−3^. The ratio of observed unique reflection is 45% and R value is over 10%. Thus, crystallographic data of **4b** can only be used for molecular structure identification as shown in Figure 1c and as the starting molecule for DFT optimization (Figure 3).

### 3.6. Computational Method

DFT calculations were performed using the Gaussian 16 program package (Gaussian, Inc., Wallingford, CT, USA) [27]. The initial geometry of the structures of **4a** and **4b** using their X-ray crystallographic data was optimized at the B3LYP/6-311G(d) level of theory. The computational time was provided by the Super Computer Laboratory, Institute for Chemical Research, Kyoto University. Frequency calculations confirmed that all the optimized geometries are the equilibrium structures. Excited energies and oscillator strengths were calculated on the optimized structures by the TD-DFT method at the B3LYP/6-311G(d) level. Tables S2, S3, S4, and S5 in the supporting information show Cartesian coordinates of the optimized geometries of **4a**, 4a-H*, **4b**, and 4b-H*, respectively.

## 4. Conclusions

In conclusion, we investigated efficient synthesis of fluorine-containing dibenzoanthracene (**4a**) and benzoperylene (**4b**) using Mallory reaction of the corresponding 1,4-distyrylbenzene-type precursor (**3**), which was prepared using addition-defluorination of available OFCP and aryllithium derivatives. The structure of **4a** originating from intermolecular π–π interactions was characterized by X-ray crystallographic analysis. In the structure of **4a**, the F–F interaction between the nearest molecules was observed. UV-Vis spectra of **4a** and **4b** in CH_2_Cl_2_ exhibited λ_max_ at 301 and 309 nm, respectively. The main PL peaks (λ_em_) of **4a** and **4b** in CH_2_Cl_2_ appeared at 411 and 418 nm, respectively, which shifted to longer wavelengths compared to those of the corresponding unsubstituted dibenzoanthracene and benzoperylene. The effect of electron-withdrawing PFCP units in **4a** and **4b** skeletons was also evaluated by computational study; TD-DFT calculation of **4a** and **4b** revealed that both HOMO and LUMO levels became lower than those of the corresponding non-fluorinated 4a-H* and 4b-H*. This finding will contribute to the design of new n-type F-PAHs.

## Figures and Tables

**Table 1:** UV-Vis and photoluminescence spectral data of **4a** and **4b** ^a^.

F-PAH	UV-Vis *^a^* λ_max_, nm (*ε*, M^−1^ cm^−1^)	Photoluminescence λ_em_, nm
**4a**	292 (47000), 301 (64000), 322 (10400), 336 (10000), 352 (11000), 380 (1120), 401 (750)	411, 433
**4b**	284 (13400), 298 (14600), 309 (17600), 379 (6600), 401 (7100)	418, 440

^a^ Measured in CH_2_Cl_2_. ^b^ [**4a**] = 1.6 × 10^−5^ M and [**4b**] = 3.1 × 10^−5^ M.

## Supplementary Materials

The following are available online. Figure S1. ^1^H NMR spectrum of **2** in CDCl_3_. Figure S2. ^19^F NMR spectrum of **2** in CDCl_3_. Figure S3. ^13^C NMR spectrum of **2** in CDCl_3_. Figure S4. ^1^H NMR spectrum of **3** in (CD_3_)_2_CO. Figure S5. ^19^F NMR spectrum of **3** in (CD_3_)_2_CO. Figure S6. ^13^C NMR spectrum of **3** in (CD_3_)_2_CO. Figure S7. ^1^H NMR spectrum of **4a** in CDCl_3_. Figure S8. ^19^F NMR spectrum of **4a** in CDCl_3_. Figure S9. ^1^H NMR spectrum of **4b** in CDCl_3_. Figure S10. ^19^F NMR spectrum of **4b** in CDCl_3_. Figure S11. ^1^H NMR spectrum of the first sublimate after sublimation of the crude product in CDCl_3_. Figure S12. UV-vis (A) and photoluminescence (B) spectra of **3** in CHCl_3_. Figure S13. Molecular structures of **3**. Figure S14. Molecular structures of **4a**. Figure S15. Molecular structure of **4b**. Table S1. Excited states and oscillator strengths of (a) dibenzoanthracene **4a** and (b). benzoperylene **4b**, calculated at B3LYP/6-611G(d) level. Table S2. Cartesian coordinates of the optimized geometry of **4a** (in Å). Table S3. Cartesian coordinates of the optimized geometry of **4a-H*** (in Å). Table S4. Cartesian coordinates of the optimized geometry of **4b** (in Å). Table S5. Cartesian coordinates of the optimized geometry of **4b-H*** (in Å).
