# Supplementary material for: Fluorine-Containing Dibenzoanthracene and Benzoperylene-Type Polycyclic Aromatic Hydrocarbons: Synthesis, Structure, and Basic Chemical Properties
Source: Molecules. 2018 Dec 16;23(12):3337. doi: 10.3390/molecules23123337 (PMC6321064; doi:10.3390/molecules23123337)
Supplement: Supplementary file 1 [file molecules-23-03337-s001.pdf]

Supplemental Materials

Article

Fluorine-Containing Dibenzoanthracene and Benzoperylene-Type Polycyclic Aromatic Hydrocarbons: Synthesis, Structure, and Basic Chemical Properties

Otohiro Gotsu [1], Tomomi Shiota [1], Hiroki Fukumoto [1,*], Tomoko Kawasaki-Takasuka [2], Takashi Yamazaki [2], Tomoko Yajima [3], Tomohiro Agou [1,*] and Toshio Kubota [1,*]

[1] Department of Quantum Beam Science, Graduate School of Science and Engineering, Ibaraki University, 4-12-1 Nakanarusawa, Hitachi, Ibaraki, 316-8511, Japan; 16nd105r@vc.ibaraki.ac.jp (O.G.); tomo.514a@gmail.com (T.S.)
[2] Division of Applied Chemistry, Institute of Engineering, Tokyo University of Agriculture and Technology, 2-24-16 Nakamachi, Koganei, Tokyo 184-8588, Japan; takasuka@cc.tuat.ac.jp (T.K.-T.); tyamazak@cc.tuat.ac.jp (T.Y.)
[3] Department of Chemistry, Faculty of Science, Ochanomizu University, Otsuka, Bunkyo-ku, Tokyo 112-8610, Japan; yajima.tomoko@ocha.ac.jp
* Correspondence: hiroki.fukumoto.chem@vc.ibaraki.ac.jp (H.F.); tomohiro.agou.mountain@vc.ibaraki.ac.jp (T.A.); toshio.kubota.organicchem@vc.ibaraki.ac.jp (T.K.); Tel.: +81-294-38-5071 (H.F.)

Contents

Figure S1. ^{1}H NMR spectrum of **2** in $CDCl_3$.

Figure S2. ^{19}F NMR spectrum of **2** in $CDCl_3$.

Figure S3. ^{13}C NMR spectrum of **2** in $CDCl_3$.

Figure S4. ^{1}H NMR spectrum of **3** in $(CD_3)_2CO$.

Figure S5. ^{19}F NMR spectrum of **3** in $(CD_3)_2CO$.

Figure S6. ^{13}C NMR spectrum of **3** in $(CD_3)_2CO$.

Figure S7. ^{1}H NMR spectrum of **4a** in $CDCl_3$.

Figure S8. ^{19}F NMR spectrum of **4a** in $CDCl_3$.

Figure S9. ^{1}H NMR spectrum of **4b** in $CDCl_3$.

Figure S10. ^{19}F NMR spectrum of **4b** in $CDCl_3$.

Figure S11. ^{1}H NMR spectrum of the first sublimate after sublimation of the crude product in $CDCl_3$.

Figure S12. UV-vis (A) and photoluminescence (B) spectra of **3** in $CHCl_3$.

Figure S13. Molecular structures of **3**.

Figure S14. Molecular structures of **4a**.

Figure S15. Molecular structure of **4b**.

Table S1. Excited states and oscillator strengths of (a) dibenzoanthracene **4a** and (b). benzoperylene **4b**, calculated at B3LYP/6-611G(d) level.

Table S2. Cartesian coordinates of the optimized geometry of **4a** (in Å).

Table S3. Cartesian coordinates of the optimized geometry of **4a-H*** (in Å).

Table S4. Cartesian coordinates of the optimized geometry of **4b** (in Å).

Table S5. Cartesian coordinates of the optimized geometry of **4b-H*** (in Å).

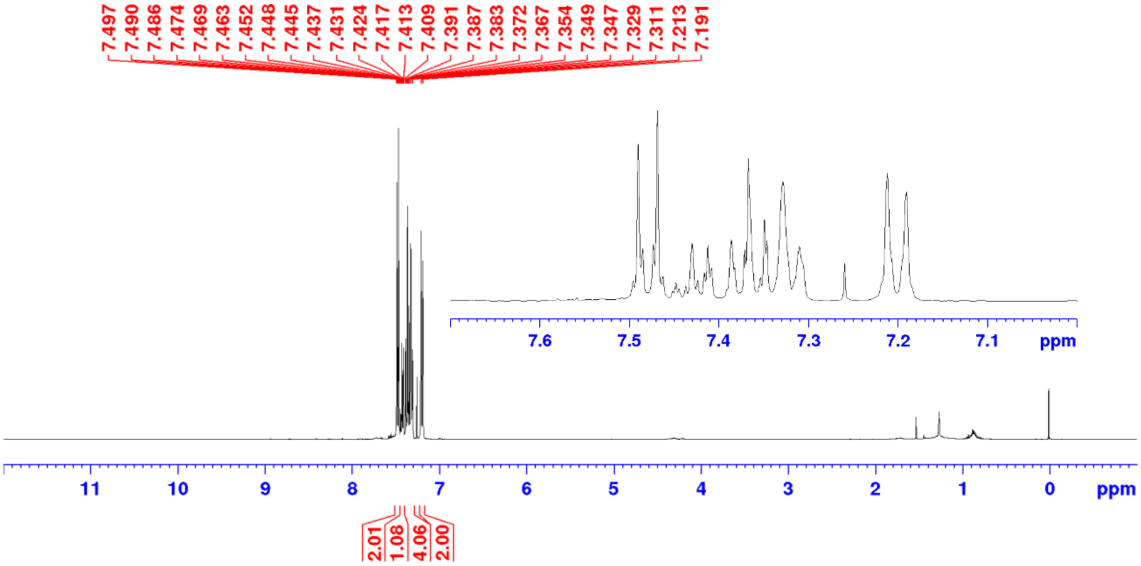

Figure S1. ^{1}H NMR spectrum of **2** in $CDCl_3$. The inset exhibits the spectrum in aromatic region.

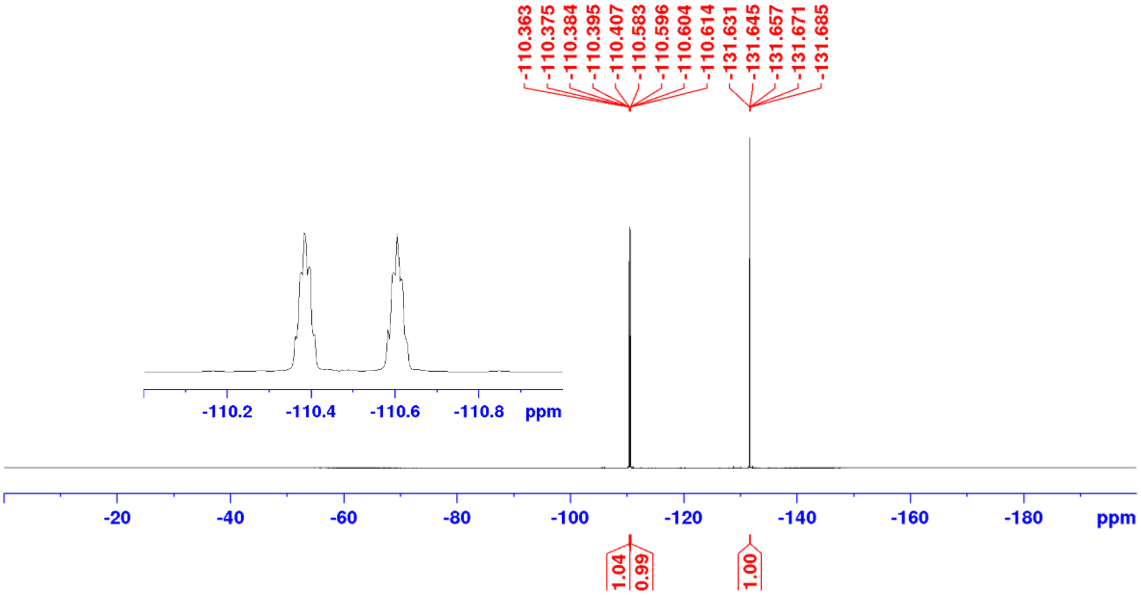

Figure S2. ^{19}F NMR spectrum of **2** in $CDCl_3$. The inset exhibits the spectrum around -111.0 ppm.

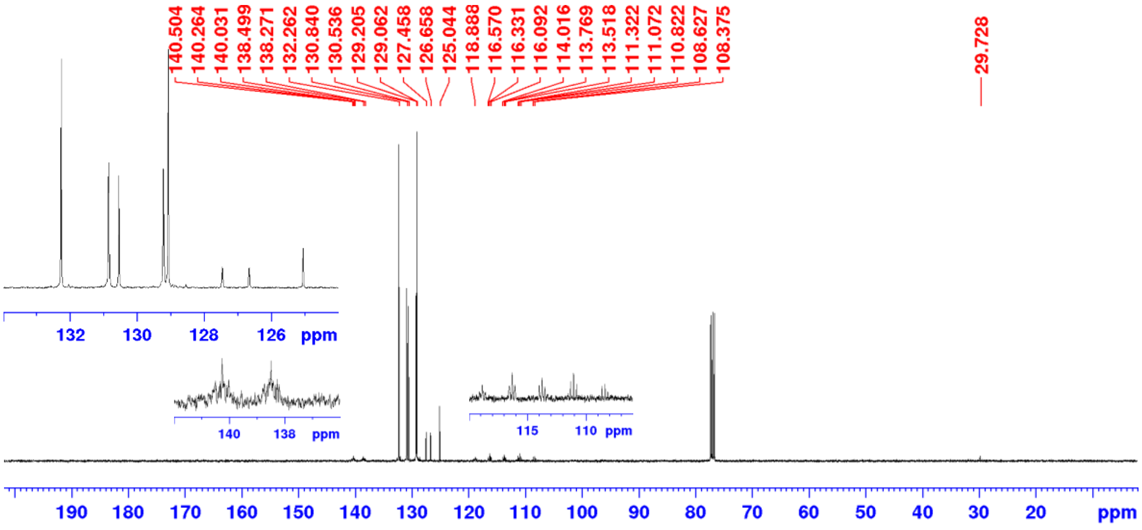

Figure S3. ^{13}C NMR spectrum of **2** in $CDCl_3$. The inset exhibits the spectra of aromatic (133–125 ppm) carbons, and the five-membered CF_2 (120–108 ppm) and sp^2 (141–138 ppm) carbons.

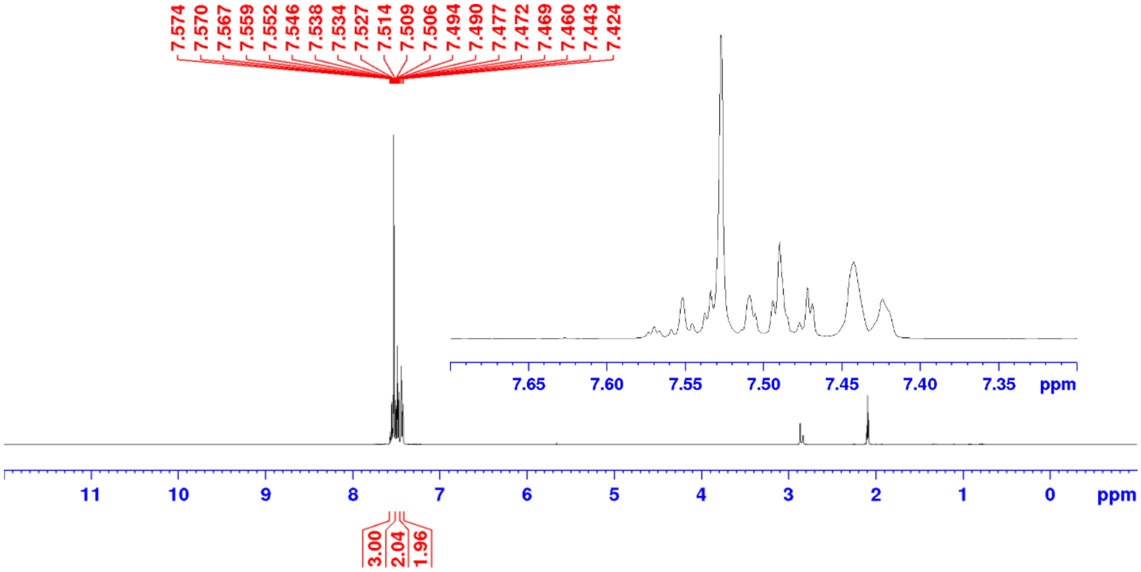

Figure S4. ^{1}H NMR spectrum of **3** in $(CD_3)_2CO$. The inset exhibits the spectrum in aromatic region.

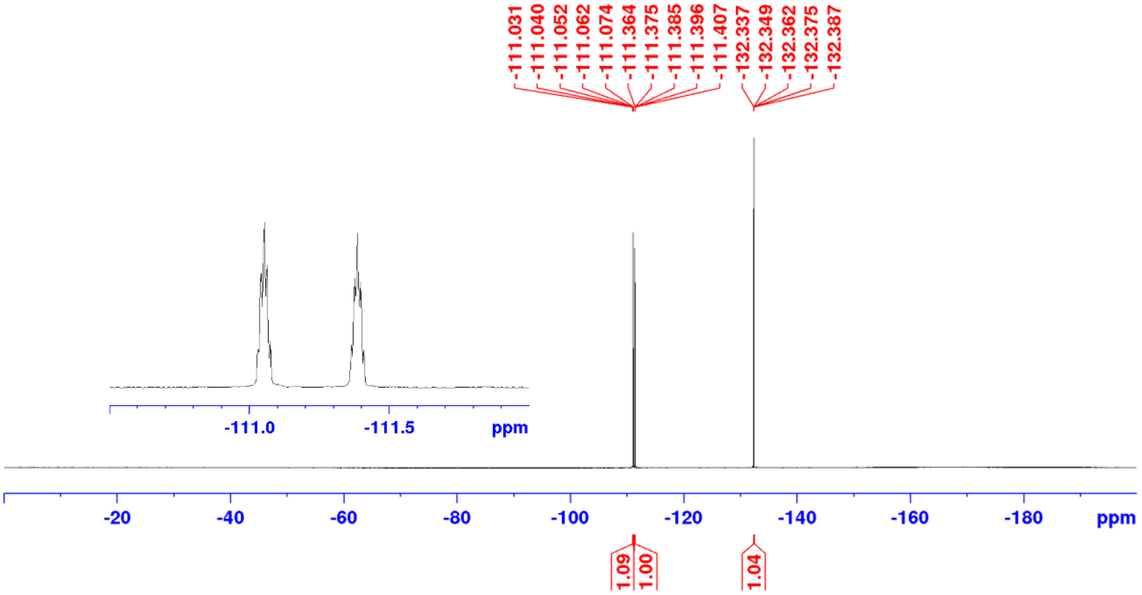

Figure S5. ^{19}F NMR spectrum of **3** in $(CD_3)_2CO$. The inset exhibits the spectrum around -111.0 ppm.

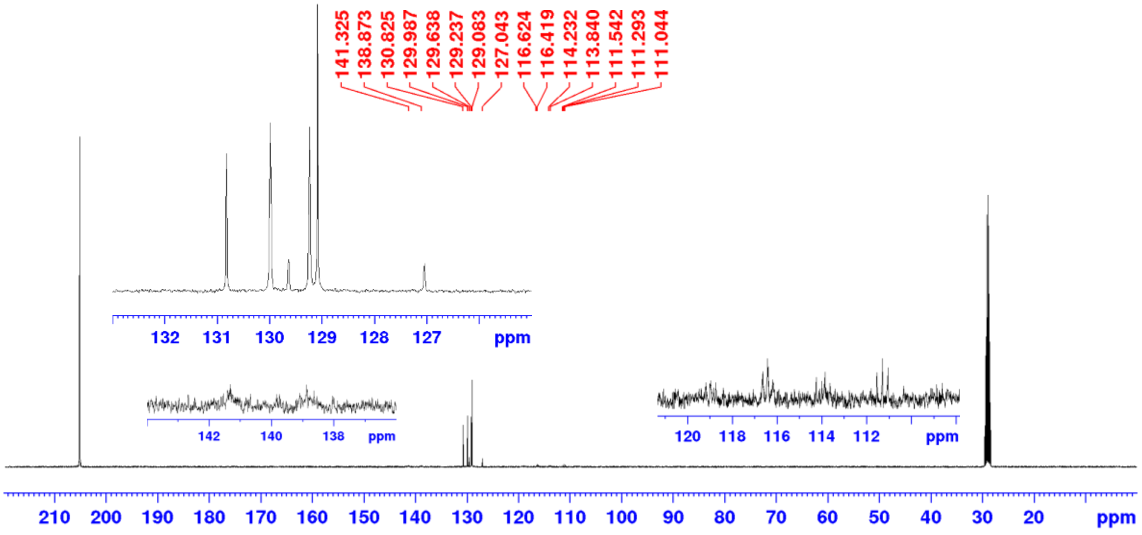

Figure S6. ^{13}C NMR spectrum of **3** in $(CD_3)_2CO$. The inset exhibits the spectra of aromatic (131– 127 ppm) carbons, and the five-membered CF_2 (120–110 ppm) and sp^2 (142–138 ppm) carbons.

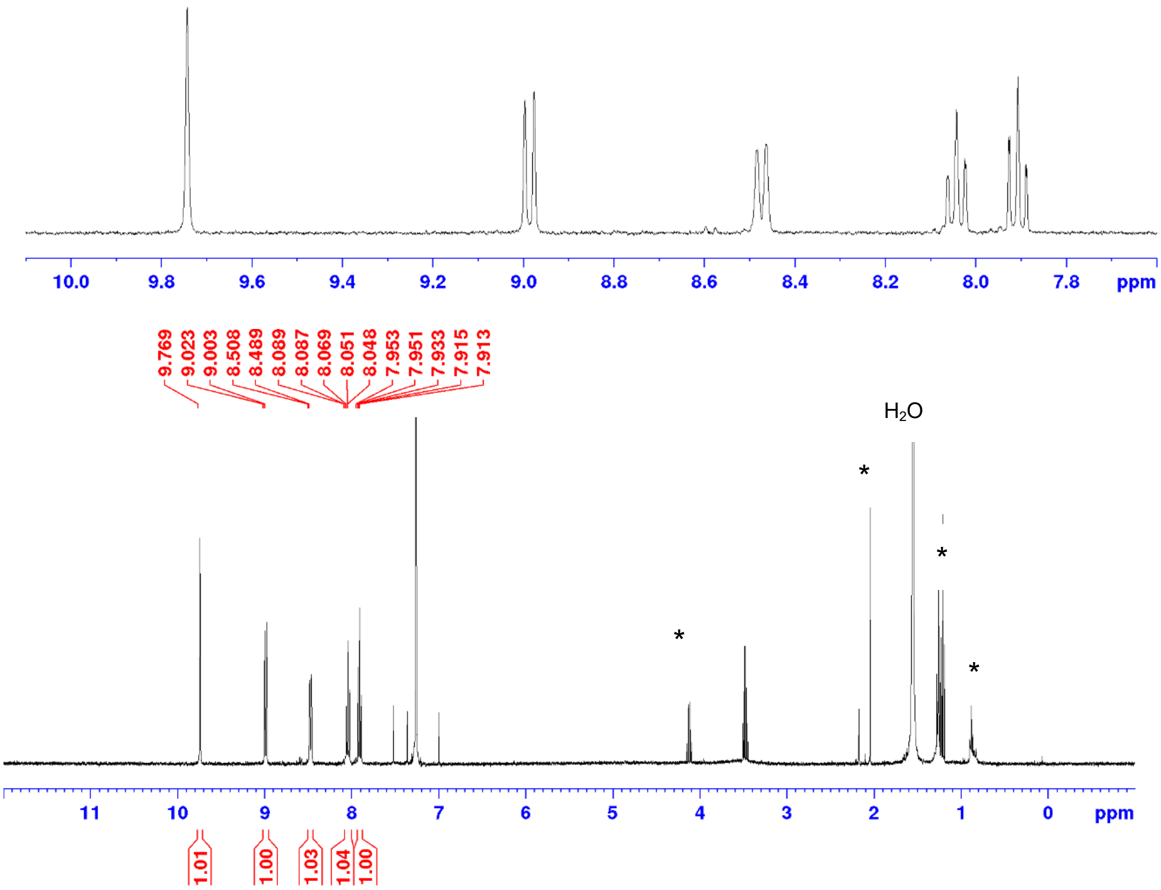

Figure S7. ^{1}H NMR spectrum of **4a** in $CDCl_3$. Asterisks (*) indicate solvent impurities (ethyl acetate and hexane) due to difficulty of removal of solvents used for purification. The upper spectrum exhibits the spectrum in the range of aromatic region.

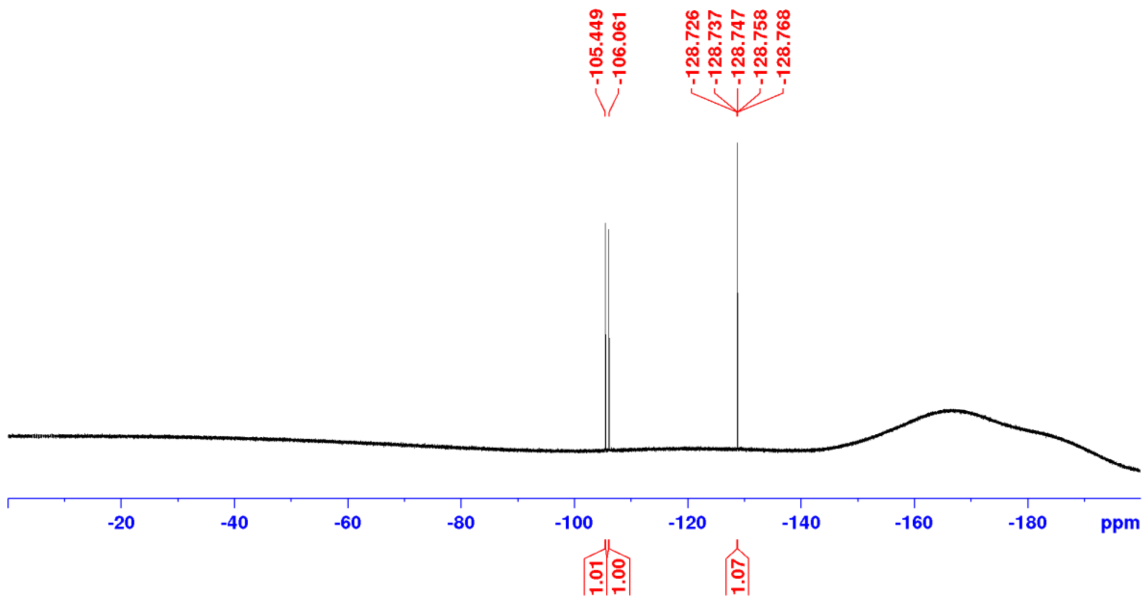

Figure S8. ^{19}F NMR spectrum of **4a** in $CDCl_3$.

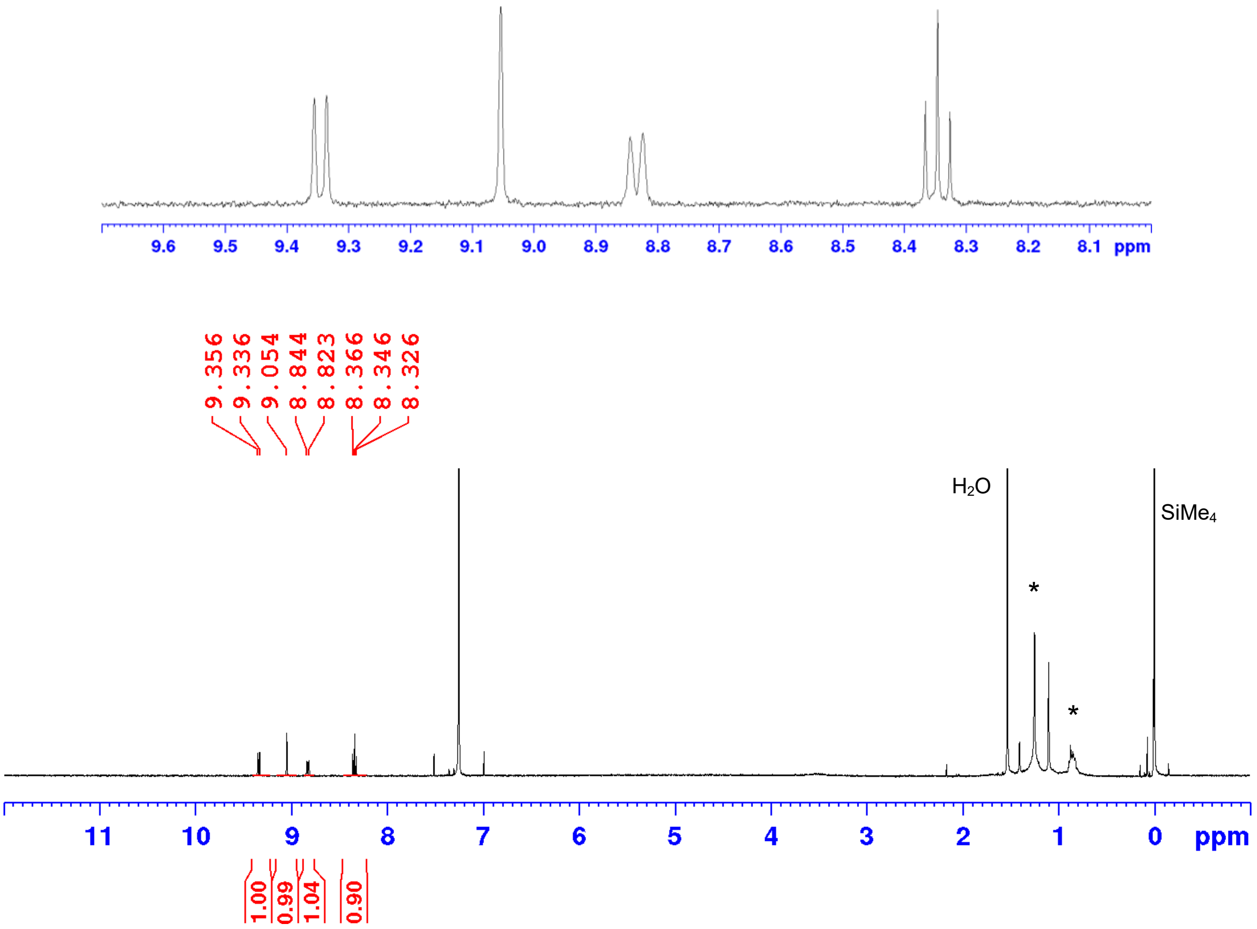

Figure S9. ^{1}H NMR spectrum of **4b** in $CDCl_3$. The upper spectrum exhibits the spectrum in the range of aromatic region. Asterisks (*) indicate solvent impurities (hexane) due to difficulty of removal of solvents used for purification.

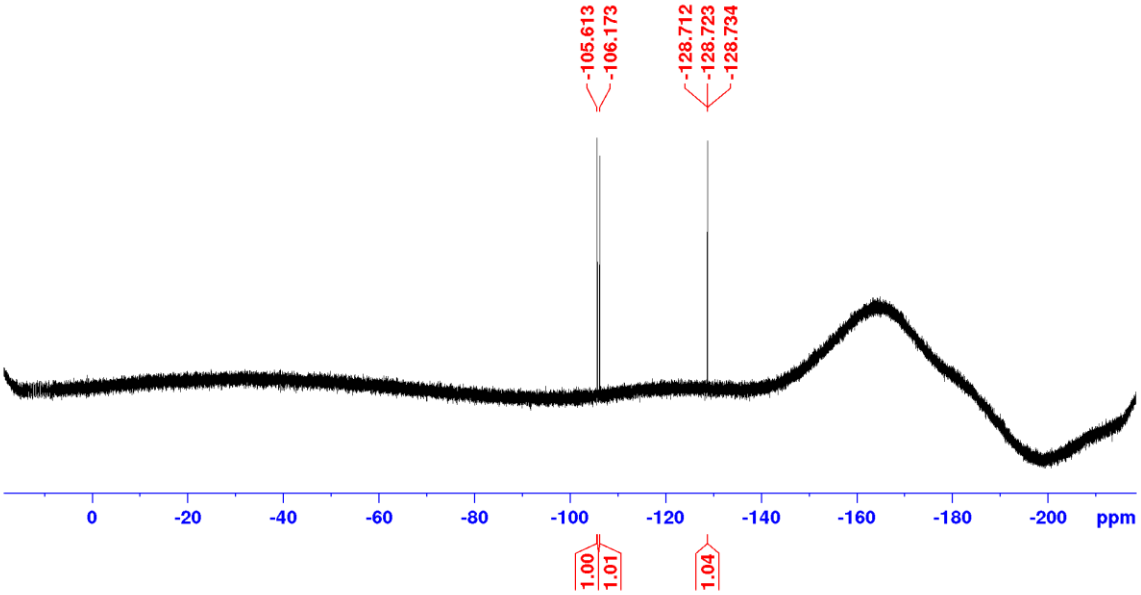

Figure S10. ^{19}F NMR spectrum of **4b** in $CDCl_3$.

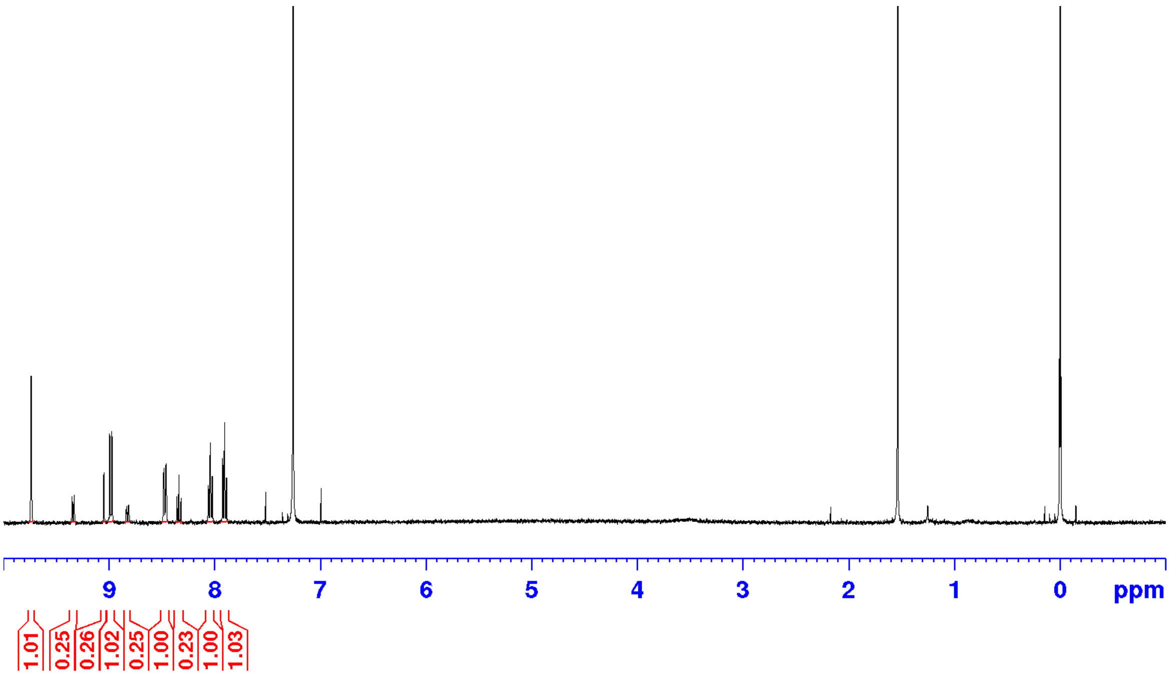

Figure S11. ^{1}H NMR spectrum of the first sublimate after sublimation of the crude product. The peaks observed in the aromatic region originate from a mixture of **4a** and **4b** (see the text and the experimental section). No peaks were observed in the aliphatic region.

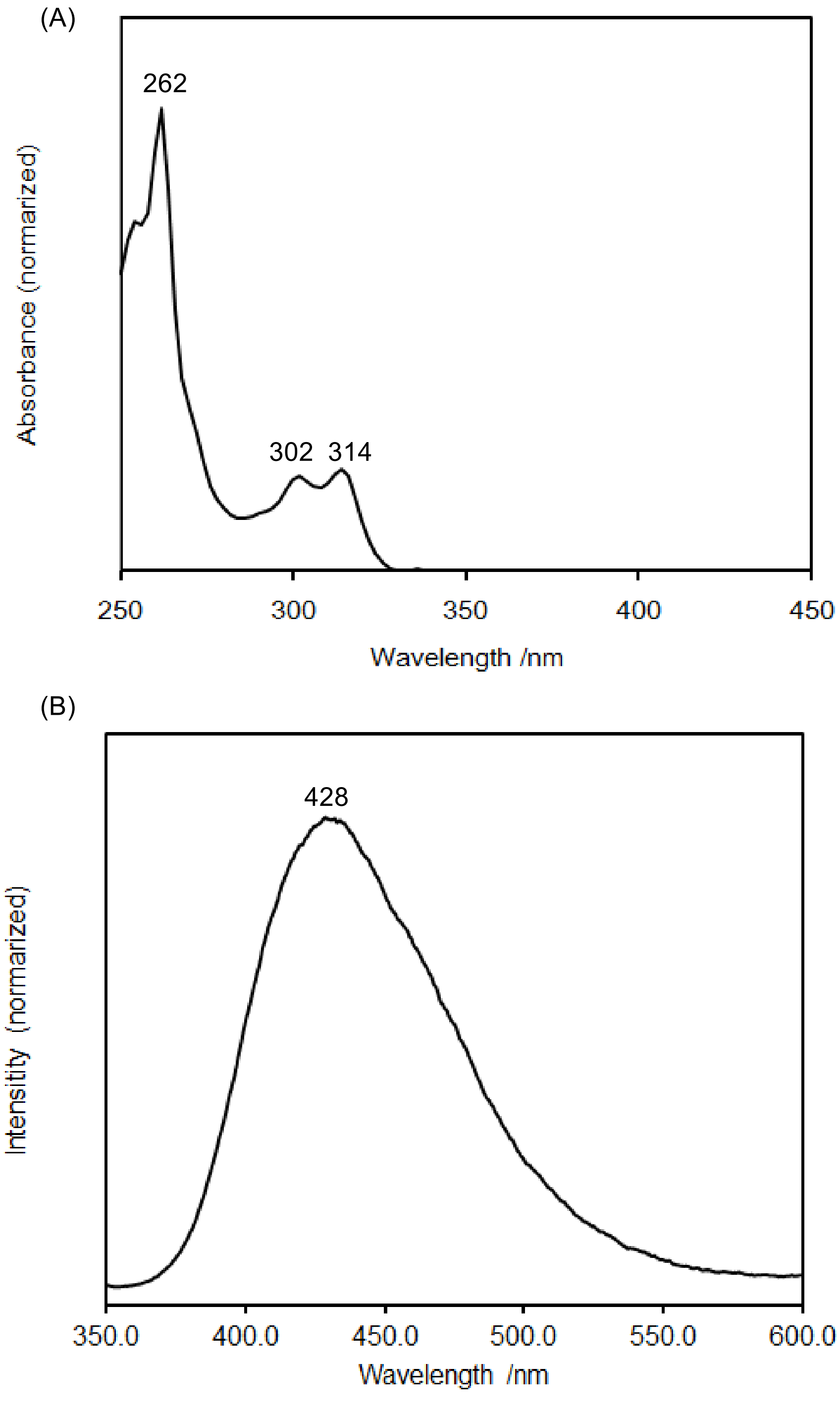

Figure S12. UV-vis (A) and photoluminescence (B) spectra of **3** in $CHCl_3$

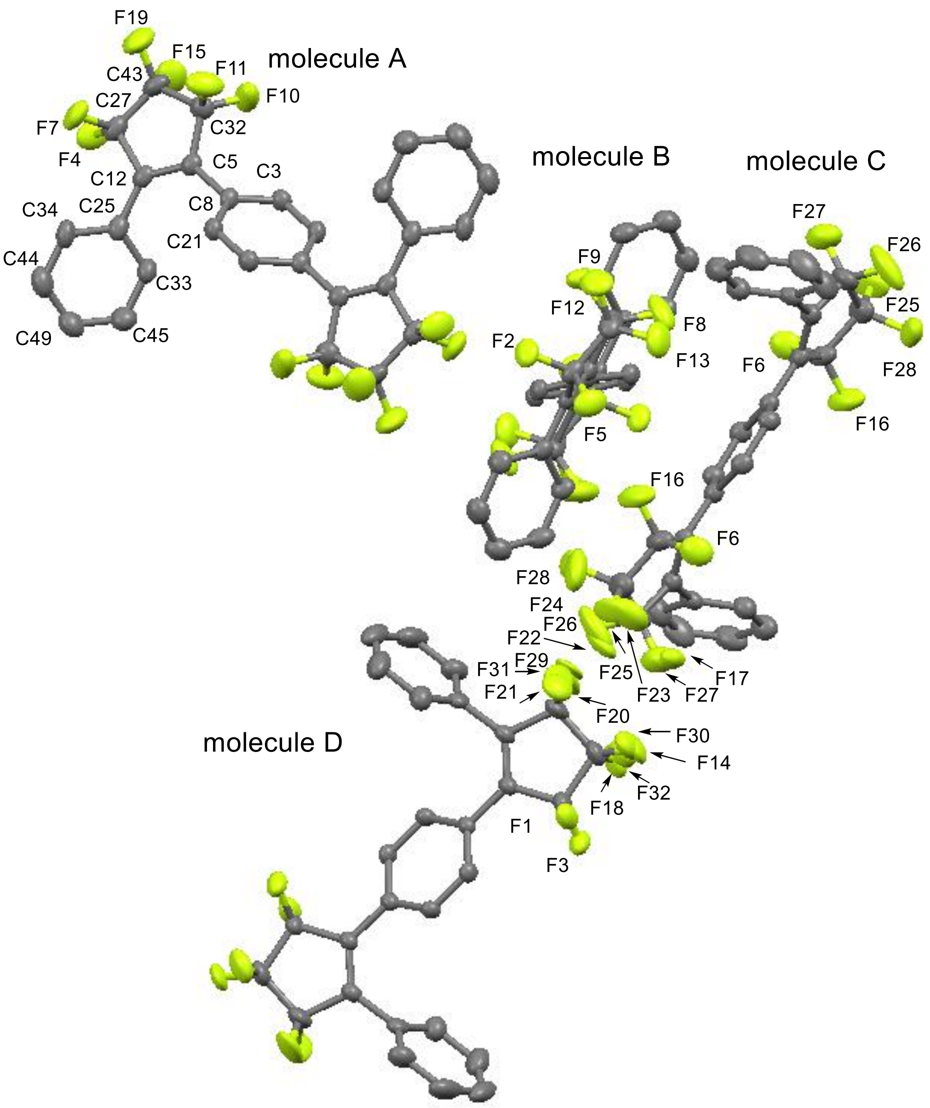

Figure S13. Molecular structures of **3**. Four independent molecules (molecules A, B, C and D) are shown. Hydrogen atoms are omitted for clarity. Fluorine atoms, F25, F26, F27 and F28 in molecule C and F14, F18, F29 and F31 in molecule D are disordered. Selected bond length (molecule A) (Å): F(4)-C(27) = 1.367(4), F(7)-C(27) = 1.351(4), F(10)-C(32) = 1.345(4), F(11)-C(32) = 1.360(4), F(15)-C(43) = 1.362(5), F(19)-C(43) = 1.324(4), C(3)-C(8) = 1.401(5), C(5)-C(12) = 1.353(5), C(5)-C(8) = 1.481(5), C(5)-C(32) = 1.496(5), C(8)-C(21) = 1.392(5), C(12)-C(25) = 1.469(5), C(12)-C(27) = 1.501(5), C(25)-C(33) = 1.390(5), C(25)-C(34) = 1.398(5), C(27)-C(43) = 1.520(6), C(32)-C(43) = 1.535(5), C(33)-C(45) = 1.379(6), C(34)-C(44) = 1.380(6), C(44)-C(49) = 1.370(6), C(45)-C(49) = 1.386(6).

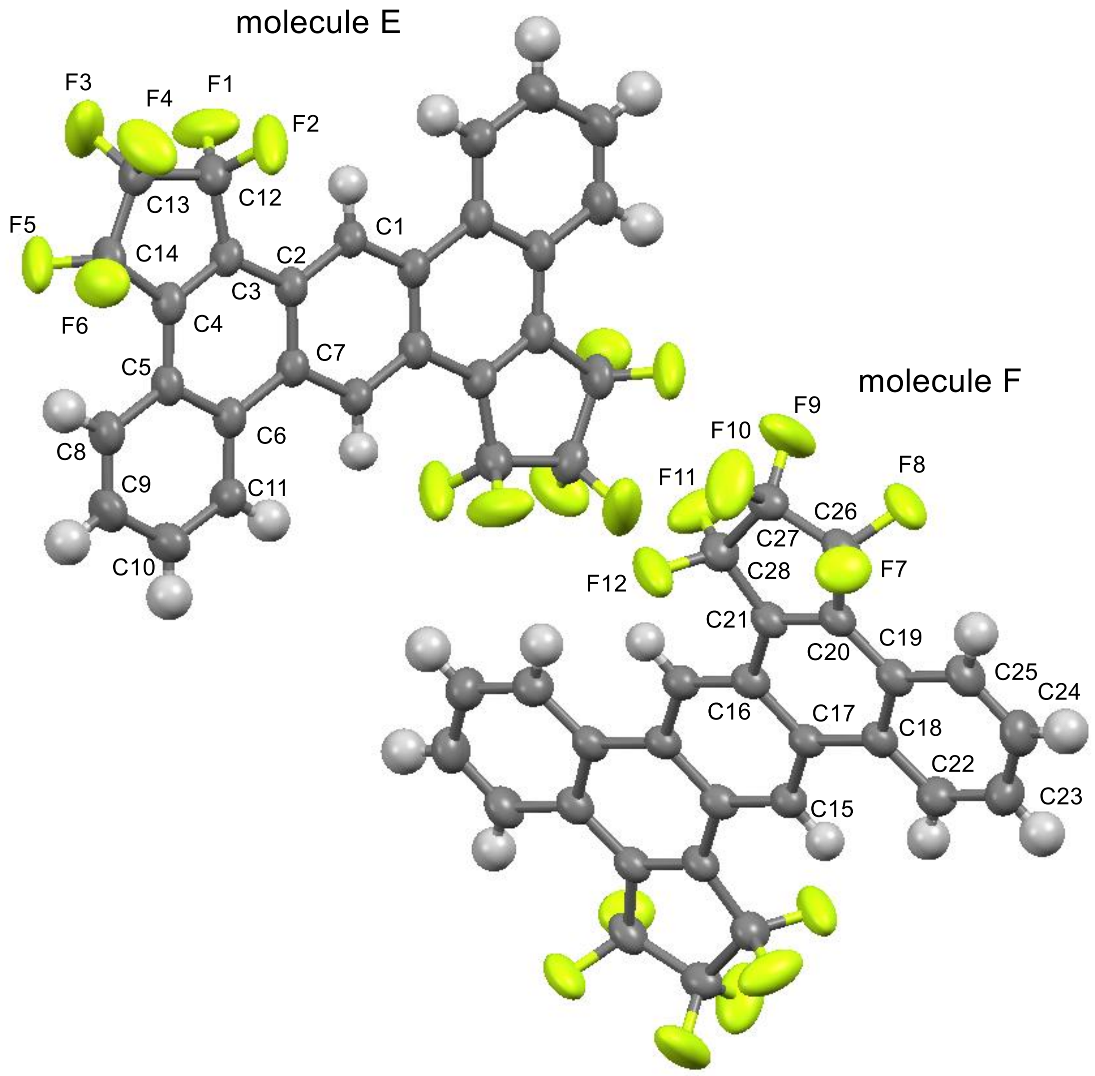

Figure S14. Molecular structures of **4a**. Two independent molecules (molecules E and F) are shown. Selected bond length (molecule E) (Å): C(1)-C(2) = 1.393(5), C(1)-C(7)* = 1.405(5), C(2)-C(7) = 1.419(5), C(2)-C(3) = 1.434(5), C(3)-C(4) = 1.354(5), C(3)-C(12) = 1.489(5), C(4)-C(5) = 1.432(5), C(4)-C(14) = 1.497(5), C(5)-C(8) = 1.411(5), C(5)-C(6) = 1.416(5), C(6)-C(11) = 1.400(5), C(6)-C(7) = 1.472(5), C(8)-C(9) = 1.357(6), C(9)-C(10) = 1.396(5), C(10)-C(11) = 1.366(5), C(12)-F(2) = 1.346(5), C(12)-F(1) = 1.349(5), C(12)-C(13) = 1.532(5), C(13)-F(4) = 1.321(4), C(13)-F(3) = 1.324(4), C(13)-C(14) = 1.542(6), C(14)-F(5) = 1.345(4), C(14)-F(6) = 1.364(4).

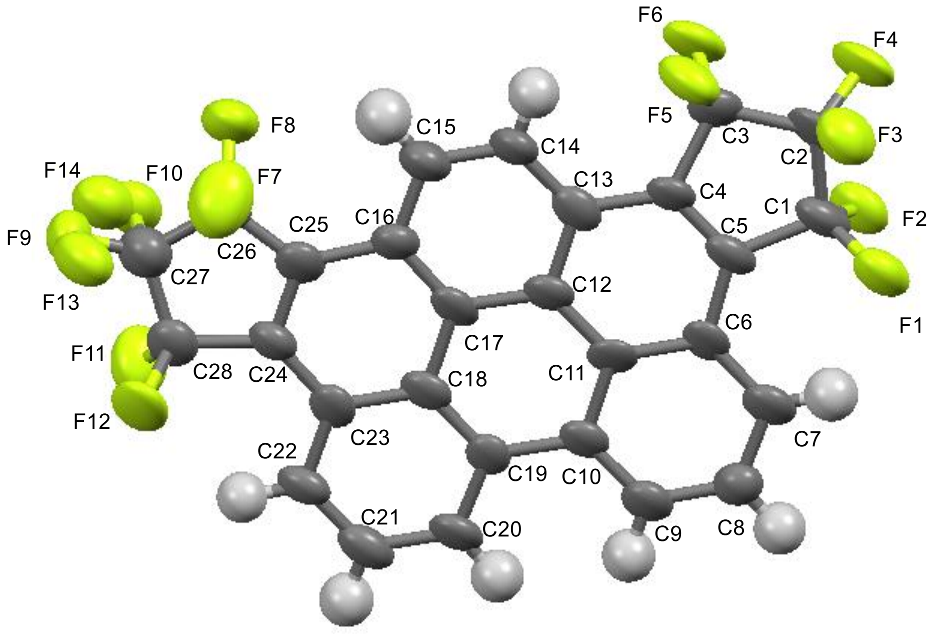

Figure S15. Molecular structure of **4b**. F1uorine atoms, F9 (F13) and F10 (F14) are disordered. For **4b**, its crystallographic data is not satisfied to discuss the bond lengths and short-contacts distances (see Section 3.5).

Table S1. Excited states and oscillator strengths of (a) dibenzoanthracene **4a** and (b) benzoperylene **4b**, calculated at B3LYP/6-611G(d) level.

(a) dibenzoanthracene-type **4a**

Excited State	Excited level	wavelength (nm)	Oscillator strengths
1	HOMO->LUMO	380.63	0.0146
2	HOMO-1->LUMO	361.61	0.1441
3	HOMO-2->LUMO	311.32	0.0000
4	HOMO->LUMO+1	305.00	0.5972
5	HOMO->LUMO+2	295.77	0.0000
6	HOMO-1->LUMO+1	290.03	0.8903
7	HOMO-3->LUMO	284.80	0.0000
8	HOMO-3->LUMO	275.98	0.0000
9	HOMO-2->LUMO+1	264.18	0.0000
10	HOMO->LUMO+3	263.41	0.0400

(b) benzoperylene-type **4b**

Excited State	Excited level	wavelength (nm)	Oscillator strengths
1	HOMO->LUMO	399.01	0.2041
2	HOMO-1->LUMO	374.66	0.007
3	HOMO->LUMO+1	306.92	0.3049
4	HOMO->LUMO+2	306.19	0.0002
5	HOMO-2->LUMO	296.79	0.0128
6	HOMO-3->LUMO	286.53	0.0094
7	HOMO->LUMO+4	283.29	0.0074
8	HOMO-1->LUMO+1	281.73	0.2882
9	HOMO-4->LUMO	269.70	0.0073
10	HOMO-1->LUMO+3	260.57	0.0324

Table S2. Cartesian coordinates of the optimized geometry of **4a** (in Å).

Symbol	X	Y	Z
H	4.533432	5.077905	-0.01514
H	2.099479	5.596849	0.037978
H	0.45213	3.808348	0.05988
H	5.284942	2.733527	-0.02639
H	-0.83612	2.321909	0.034896
H	-4.53343	-5.07791	0.015132
H	-2.09948	-5.59685	-0.03798
H	-0.45213	-3.80835	-0.05988
H	-5.28494	-2.73353	0.026387
H	0.836122	-2.32191	-0.0349
C	3.805387	4.274229	-0.00209
C	1.894776	2.192184	0.025112
C	0.925314	1.095095	0.019882
C	2.434597	4.565144	0.027053
C	1.501964	3.5457	0.040166
C	3.290386	1.906064	0.003589
C	4.227253	2.960767	-0.01164
C	-0.45267	1.313146	0.024105
C	1.375936	-0.26324	-0.00488
C	3.477612	-1.82933	-0.09958
C	5.102921	0.011969	-0.09331
C	2.793218	-0.49582	-0.0134
C	3.692673	0.527052	-0.01016
C	4.970457	-1.51064	0.204671
C	-3.80539	-4.27423	0.002087
C	-1.89478	-2.19218	-0.02511
C	-0.92531	-1.0951	-0.01988
C	-2.4346	-4.56515	-0.02706
C	-1.50196	-3.5457	-0.04017
C	-3.29039	-1.90606	-0.00359
C	-4.22725	-2.96077	0.011637
C	0.452674	-1.31315	-0.02411
C	-1.37594	0.263236	0.004879
C	-3.47761	1.829327	0.099575
C	-5.10292	-0.01197	0.093309
C	-2.79322	0.495821	0.013403
C	-3.69267	-0.52705	0.010159
C	-4.97046	1.510643	-0.20466
F	5.818502	-2.25748	-0.52381
F	2.996331	-2.75504	0.778951
F	5.63537	0.198016	-1.3363
F	5.957283	0.599617	0.789052
F	3.361142	-2.38154	-1.34336
F	5.216414	-1.73311	1.515879
F	-5.8185	2.257478	0.523845
F	-2.99633	2.755031	-0.77896
F	-5.63538	-0.19803	1.336295
F	-5.95728	-0.59961	-0.78907
F	-3.36113	2.381543	1.343352
F	-5.21643	1.733132	-1.51586

Table S3. Cartesian coordinates of the optimized geometry of **4a-H*** (in Å).

Symbol	X	Y	Z
H	2.712174	6.243689	-0.12743
C	2.29131	5.243756	-0.09668
C	3.134597	4.124146	-0.0831
H	4.211934	4.254374	-0.1017
C	0.922171	5.066829	-0.07053
C	2.598415	2.850318	-0.04832
C	1.205341	2.633441	-0.02468
C	0.352505	3.776268	-0.03108
C	0.614663	1.295398	-0.00346
C	-0.81132	1.152628	0.006256
C	-1.6235	2.337335	0.017942
C	-1.07134	3.581345	0.001361
C	-2.13357	4.657661	-0.02824
C	-3.1346	2.403473	0.000862
C	-3.4299	3.888323	0.323776
C	-1.37887	-0.1268	0.001206
C	-0.61466	-1.2954	-0.00346
C	0.811319	-1.15263	0.006256
C	1.378873	0.126796	0.001206
C	1.623499	-2.33734	0.017942
C	1.07134	-3.58135	0.001361
C	-0.35251	-3.77627	-0.03108
C	-1.20534	-2.63344	-0.02468
C	-2.59842	-2.85032	-0.04832
C	-3.1346	-4.12415	-0.0831
C	-2.29131	-5.24376	-0.09668
C	-0.92217	-5.06683	-0.07053
C	3.134597	-2.40347	0.000862
C	3.429898	-3.88832	0.323776
C	2.133571	-4.65766	-0.02824
H	0.267766	5.931804	-0.08279
H	3.277743	2.006484	-0.04243
H	-2.45911	-0.19305	-0.00326
H	-3.27774	-2.00648	-0.04243
H	-4.21193	-4.25437	-0.1017
H	-2.71217	-6.24369	-0.12743
H	-0.26777	-5.9318	-0.08279
H	2.459113	0.193046	-0.00326
H	1.938275	-5.47502	0.672822
H	3.632564	-3.99213	1.393357
H	4.304493	-4.26973	-0.20757
H	3.603943	-1.72608	0.721125
H	-3.60394	1.72608	0.721125
H	-4.30449	4.269734	-0.20757
H	-3.63256	3.99213	1.393357
H	-2.18971	5.113258	-1.02609
H	-1.93828	5.475022	0.672822
H	-3.51889	2.118937	-0.98792
H	3.518894	-2.11894	-0.98792
H	2.189714	-5.11326	-1.02609

Table S4. Cartesian coordinates of the optimized geometry of **4b** (in Å).

Symbol	X	Y	Z
H	4.646869	2.81142	0.106716
H	3.409965	4.947343	0.14561
H	0.976249	4.976483	0.085854
H	1.221291	-3.04257	-0.00794
H	-1.22129	-3.04257	0.007953
H	-0.97625	4.976483	-0.08585
H	-3.40996	4.947344	-0.14561
H	-4.64687	2.811423	-0.10672
C	5.008809	0.091902	0.107044
C	5.16783	-1.418	-0.23942
C	3.76694	-2.02761	0.062446
C	2.837338	-0.848	0.008192
C	3.526513	0.329045	0.031474
C	2.86445	1.597421	0.045182
C	3.565276	2.812754	0.08893
C	2.869429	4.00795	0.105938
C	1.477675	4.017597	0.070045
C	0.732694	2.834921	0.021865
C	1.434123	1.59505	0.019594
C	0.71442	0.351546	0.002799
C	1.408035	-0.88865	0.000136
C	0.685484	-2.10288	-0.00175
C	-0.68549	-2.10288	0.001756
C	-1.40804	-0.88865	-0.00013
C	-0.71442	0.351546	-0.0028
C	-1.43412	1.595051	-0.01959
C	-0.73269	2.834921	-0.02186
C	-1.47767	4.017598	-0.07004
C	-2.86943	4.007951	-0.10594
C	-3.56528	2.812756	-0.08893
C	-2.86445	1.597423	-0.04518
C	-3.52651	0.329046	-0.03147
C	-2.83734	-0.848	-0.00819
C	-3.76694	-2.02761	-0.06244
C	-5.16783	-1.418	0.239404
C	-5.00881	0.091904	-0.10705
F	5.734572	0.861149	-0.75062
F	5.495987	0.335908	1.358626
F	5.434892	-1.5464	-1.55919
F	6.152624	-2.00943	0.458322
F	3.465615	-3.00735	-0.83366
F	3.769952	-2.61288	1.29549
F	-3.76994	-2.6129	-1.29548
F	-3.46562	-3.00734	0.833681
F	-6.15262	-2.00943	-0.45835
F	-5.43492	-1.5464	1.559171
F	-5.73457	0.861147	0.750623
F	-5.49598	0.335918	-1.35863

Table S5. Cartesian coordinates of the optimized geometry of **4b-H*** (in Å).

Symbol	X	Y	Z
C	0.195988	-1.41954	0.915812
C	0.093235	-0.7076	-0.32525
C	-0.09324	0.7076	-0.32525
C	-0.19599	1.419543	0.915812
C	-0.10811	0.725894	2.158154
C	0.108106	-0.72589	2.158154
C	0.181886	-1.40643	-1.56067
C	0.362326	-2.82529	-1.53428
C	0.464009	-3.50834	-0.35762
C	0.395204	-2.83736	0.905599
C	-0.18189	1.406426	-1.56067
C	-0.08852	0.681061	-2.77045
C	0.516937	-3.52521	2.123854
C	0.438882	-2.84043	3.324635
C	0.235012	-1.46278	3.34121
C	0.088518	-0.68106	-2.77045
C	-0.36233	2.825291	-1.53428
C	-0.46401	3.508339	-0.35762
C	-0.3952	2.837357	0.905599
C	-0.23501	1.462778	3.34121
C	-0.43888	2.840433	3.324635
C	-0.51694	3.525213	2.123854
C	-0.51089	3.750134	-2.72244
C	-0.36233	5.158326	-2.09685
C	-0.69245	4.984432	-0.59408
C	0.510889	-3.75013	-2.72244
C	0.362326	-5.15833	-2.09685
C	0.692452	-4.98443	-0.59408
H	-0.16066	1.212904	-3.71302
H	0.160662	-1.2129	-3.71302
H	-0.53617	3.377161	4.263104
H	-0.67619	4.597844	2.119998
H	-0.18006	0.966424	4.301725
H	0.180063	-0.96642	4.301725
H	0.676186	-4.59784	2.119998
H	0.536172	-3.37716	4.263104
H	-0.07011	5.623479	0.039884
H	-1.73494	5.251601	-0.37456
H	0.673629	5.492417	-2.20242
H	-0.99262	5.905421	-2.58402
H	-1.49555	3.616391	-3.19001
H	0.230566	3.568318	-3.50641
H	-0.23057	-3.56832	-3.50641
H	1.495552	-3.61639	-3.19001
H	-0.67363	-5.49242	-2.20242
H	0.992616	-5.90542	-2.58402
H	1.734935	-5.2516	-0.37456
H	0.070108	-5.62348	0.039884
